# Acute Care Utilization Patterns During Chemotherapy and Predictive Model Development at a Rural Community Cancer Center

**DOI:** 10.1200/CCI-25-00186

**Published:** 2025-11-13

**Authors:** McKenna Perrin, Crystal Hattum, Jamie Arens, Tobias Meissner

**Affiliations:** ^1^ Center for Precision Oncology, Avera Cancer Institute, Sioux Falls, SD

## Abstract

**PURPOSE:**

Acute care use (ACU) is more costly and prolonged for oncology patients and often leads to treatment disruptions and worsened outcomes. Reducing ACU requires understanding risk factors and proactively identifying at-risk patients. This study addresses research gaps by developing predictive models to assess all-cause acute care use (A-ACU) versus preventable acute care use (P-ACU) and rural-specific barriers.

**PATIENTS AND METHODS:**

We conducted a retrospective cohort study of adult oncology patients who received intravenous cancer treatment between October 2021 and April 2024 within a rural midwestern regional cancer network. We used predictor and outcome data from electronic medical records and insurance claims. We defined P-ACU using the Centers for Medicare & Medicaid Services' OP-35 criteria and classified A-ACU as any emergency department visit or hospitalization, regardless of reason. We trained LASSO and Random Forest models on 80% of the cohort to predict 30-, 90-, and 180-day risk of P-ACU and A-ACU after regimen initiation.

**RESULTS:**

Among 2,922 patients, 45.3% experienced A-ACU and 10.3% had P-ACU within 180 days of chemotherapy regimen initiation. Key predictors included number of previous inpatient stays and comorbidities. Insurance type and age were more influential in predicting P-ACU, whereas laboratory values (albumin, sodium, and neutrophil-to-lymphocyte ratio) were more important in A-ACU models. Nearly all LASSO and Random Forest models showed strong performance (mean area under the receiver operating characteristic curve = 0.73, mean F1 score = 0.79).

**CONCLUSION:**

Our models effectively identify patients at high risk for ACU using routinely collected data and validate known risk factors in a large rural oncology population. Future work should integrate these tools into practice and address rural-specific challenges to reduce ACU during chemotherapy.

## INTRODUCTION

Despite progress in reducing mortality, the burden of cancer in the United States continues to grow. Driven largely by an aging and expanding population, 2024 was the first year projected to reach over 2 million new cancer cases.^1^ As cancer diagnoses rise, so too does the number of people receiving chemotherapy.^2^ Despite the survival benefits of chemotherapy, treatment can result in adverse events requiring acute care use (ACU), including emergency department (ED) visits or hospitalizations.^3^ Over half of the chemotherapy patients become hospitalized^3,4^ and average one chemotherapy-related ED visit annually.^5^ In the United States, hospitalizations for cancer-related purposes are, on average, 1.5 days longer and incur a cost of $22,100—almost double the cost of non–cancer-related hospitalizations.^6^ Similarly, patients with cancer experience higher ED expenses with the median visit costing $1,047 compared with $335 for those without cancer.^7^ The financial burden is further exacerbated by the higher likelihood of ED visits in oncology patients leading to inpatient hospitalization compared with nononcology patients.^8^ Additionally, ACU may put oncology patients at risk for complications, disrupt treatment regimens, and negatively affect patient experiences.^4,9^

**CONTEXT**

**Key Objective**
Can predictive models built on routinely collected data identify oncology patients at high risk for preventable and all-cause acute care utilization, and highlight distinct risk factors in a rural setting?
**Knowledge Generated**
This study developed and validated models showing strong predictive performance in identifying patients at risk for preventable and all-cause acute care utilization, with comorbidity burden, previous hospitalizations, and inflammatory/nutritional biomarkers as key predictors. Risk factor importance varied by outcome, with insurance and age more influential in preventable acute care utilization prediction and laboratory values more important for all-cause acute care utilization.
**Relevance *(P.P. Yu)***
Logistic regression and random forest predictive models of postchemotherapy emergency department and hospitalization risk were developed and retrospectively evaluated in rural and urban populations within the same health care system.*
*Relevance section written by *JCO Clinical Cancer Informatics* Associate Editor Peter Paul Yu, MD, FACP, FASCO.

Among oncology patients experiencing ACU, an estimated 51.6% of these visits may be considered preventable.^9^ The Centers for Medicare & Medicaid Services (CMS) defines preventable acute care use (P-ACU) as an ED visit or hospitalization linked to an anemia, dehydration, diarrhea, emesis, fever, nausea, neutropenia, pain, pneumonia, or sepsis diagnosis.^10^ To track 30-day P-ACU rates after chemotherapy, CMS introduced Chemotherapy Measure OP-35.^10^ Measuring ACU rates provides insights for tracking facility performance and comparing outcomes across institutions, but incorporating predictive strategies and identifying modifiable risk factors is necessary to drive reduction. However, disparities persist in cancer care access and outcomes, particularly between rural and urban populations. Rural patients often face challenges such as limited access to specialized oncology care.^11,12^ These challenges compounded by factors such as geographic distance, transportation barriers, and socioeconomic factors can lead to delayed diagnosis, more advanced disease presentation, and increased reliance on ED services.^13,14^

Existing research on ACU in oncology patients underscores the need for larger, more diverse data sets to identify modifiable risk factors and effective interventions.^15^ Many models find mixed significance of associations with potential predictors.^4,15-23^ Additionally, few models have compared factors differentially affecting P-ACU versus all-cause acute care use (A-ACU), examined different time windows for ACU, or specifically addressed challenges faced by rural patients. In response to these gaps, we developed predictive models to estimate both A-ACU and P-ACU utilization at 30, 90, and 180 days after cancer treatment initiation. This research may help validate previously reported associations, offer insights on P-ACU versus A-ACU risk, and identify unique risk factors facing rural patients.

## PATIENTS AND METHODS

### Study Design

This retrospective cohort study was approved by the institutional review board with a waiver of informed consent (#2023.081). The study was conducted in accordance with the protocol, International Conference on Harmonisation, Good Clinical Practice guidelines, applicable regulations, and ethical principles according to Declaration of Helsinki.

### Setting and Time Frame

We conducted this study using electronic medical record (EMR) data from cancer treatment visits within a nationally accredited cancer network. This system has six cancer centers and serves patients across a 72,000-square-mile region spanning South Dakota, Iowa, Minnesota, and Nebraska. For this retrospective analysis, initial intravenous cancer treatment regimen infusions of a regimen given between October 1, 2021, and May 1, 2024, were considered. If a patient had multiple qualifying initial infusions during this period, only the first was included. ACU data were collected through October 29, 2024, to provide a full 180 days of follow-up.

### Study Population

**FIG 1.**
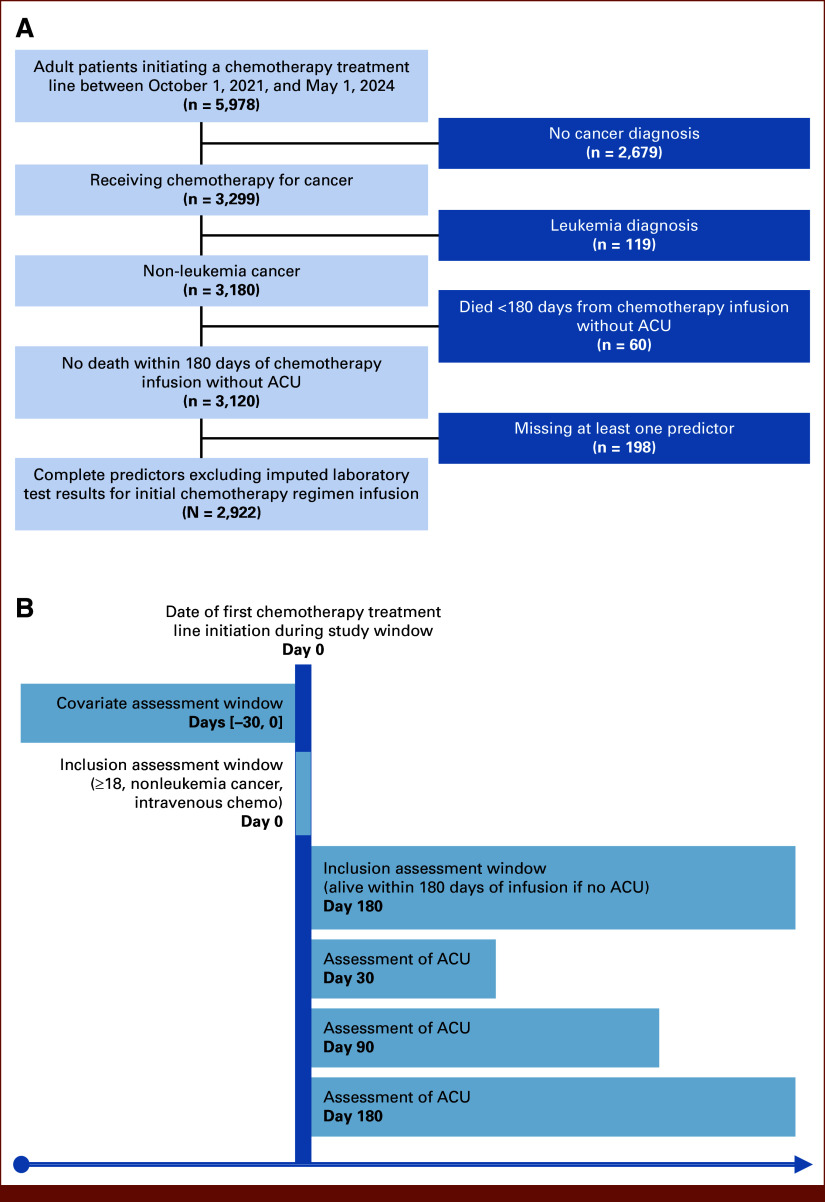
(A) Flow diagram illustrating the process of patient inclusion in the study, N = 2,922. (B) Diagram depicting the study design, including covariate assessment window, inclusion criteria, cohort entry point, and outcome follow-up period. ACU, acute care use.

Patients were eligible for inclusion in the study if they met the following criteria: (1) age 18 years and older at the time of the cancer treatment and (2) had a nonleukemia cancer diagnosis, consistent with CMS OP-35 measure requirements. Additional criteria included (3) initiation of an intravenous cancer treatment regimen and (4) survival for ≥180 days after regimen initiation if no ACU event occurred. A total of 3,120 patients met inclusion criteria. Of these, 2,922 with complete predictors were eligible for model development (Fig 1A).

### Predictors

On the basis of literature review (Data Supplement, Table S1) and stakeholder input, covariates were queried from two EMR systems: MEDITECH Expanse (Medical Information Technology, Inc, Westwood, MA) and MOSAIQ (Elekta, Stockholm, Sweden). MEDITECH Expanse, the primary hospital-wide EMR, provided demographics, comorbidities, laboratory values, and health care utilization. MOSAIQ, an oncology-specific EMR used to manage cancer treatment, provided cancer treatment (section Treatment Categorization) and clinician-assessed performance status (Eastern Cooperative Oncology Group [ECOG]). Extracted variables included age at infusion, body system of cancer diagnosis, laboratory values (neutrophil-to-lymphocyte ratio [NLR], albumin, and sodium), cancer treatment modality, smoking status, Charlson comorbidity index,^24^ ECOG, and number of inpatient admissions within 6 months preceding treatment. Patient home addresses were geocoded and linked to Rural-Urban Commuting Area (RUCA)^25^ codes to classify rurality. Missing numeric values (ECOG, 10% [n = 298]; NLR, 13% [n = 389]; albumin, 1% [n = 35]; sodium, 1% [n = 32]) were imputed using k-nearest neighbors. Predictors reflected the most recent value before regimen initiation, taken at most 30 days before initial infusion (Fig 1B). Table 1 summarizes patient characteristics stratified by the occurrence of ACU within 180 days of regimen initiation: those having P-ACU, A-ACU without P-ACU, no ACU, and the overall cohort.

**TABLE 1.** Characteristics of the Study Population Stratified by ACU Occurrence: P-ACU Within ≤180 Days of Chemotherapy Initiation, A-ACU Within ≤180 Days Without P-ACU, and No ACU

Characteristic	N = 2,922	n = 302	n = 1,033	n = 1,587	*P*
	Overall	P-ACU Within ≤180 days	A-ACU Within ≤180 days	No ACU Within ≤180 days	
Demographics					
Age, years, median (IQR)	68 (59-75)	72 (65-79)	67 (58-74)	67 (58-74)	<.001
Female, No. (%)	1,524 (52)	143 (47)	520 (50)	861 (54)	.031
Race, No. (%)					.002
African American	28 (1.0)	1 (0.3)	16 (1.6)	11 (0.7)	
Asian	20 (0.7)	5 (1.7)	4 (0.4)	11 (0.7)	
Caucasian	2,727 (94)	289 (96)	949 (93)	1,489 (95)	
Native American	63 (2.2)	2 (0.7)	34 (3.3)	27 (1.7)	
Other	49 (1.7)	3 (1.0)	17 (1.7)	29 (1.9)	
Unknown	35	2	13	20	
Ethnicity, No. (%)					.8
Hispanic	0 (0)	1 (0.2)	1 (0.2)	1 (0.1)	
Not Hispanic/Latino	143 (100)	414 (100)	414 (100)	766 (100)	
Unknown	159	618	618	820	
Insurance, No. (%)					<.001
Commercial	1,168 (40)	56 (19)	439 (42)	673 (42)	
Medicaid	122 (4.2)	5 (1.7)	61 (5.9)	56 (3.5)	
Medicare	1,494 (51)	233 (77)	480 (46)	781 (49)	
Self-pay	93 (3.2)	7 (2.3)	30 (2.9)	56 (3.5)	
Other	45 (1.5)	1 (0.3)	21 (2.3)	21 (1.3)	
RUCA categorization, No. (%)					.6
Metropolitan	857 (29)	98 (32)	287 (28)	472 (30)	
Micropolitan	909 (31)	90 (30)	339 (33)	480 (30)	
Small town	319 (11)	32 (11)	118 (11)	169 (11)	
Rural	837 (29)	82 (27)	289 (28)	466 (29)	
Comorbidities					
Weighted Charlson comorbidity index, No. (%)					<.001
≤2	922 (32)	42 (14)	275 (27)	605 (38)	
3-4	682 (23)	87 (29)	230 (22)	365 (23)	
5-7	978 (33)	126 (42)	371 (36)	481 (30)	
≥8	340 (12)	47 (16)	157 (15)	136 (8.6)	
Previous inpatient admissions, No. (%)					<.001
0	2,042 (70)	185 (61)	632 (61)	1,225 (77)	
1	628 (21)	74 (25)	269 (26)	285 (18)	
≥2	252 (8.6)	43 (14)	132 (13)	77 (4.9)	
ECOG, No. (%)					<.001
0	1,563 (53)	120 (40)	490 (47)	953 (60)	
1	1,150 (39)	154 (51)	438 (42)	558 (35)	
≥2	209 (7.2)	28 (9.3)	105 (10)	76 (4.8)	
Smoking status, No. (%)					.012
Current smoker	361 (12)	29 (9.6)	133 (13)	199 (13)	
Former smoker	1,114 (38)	144 (48)	423 (41)	547 (34)	
Never smoker	1,447 (50)	129 (43)	477 (46)	841 (53)	
Laboratory values					
Neutrophil/lymphocyte ratio, median (IQR)	3.1 (2.1-4.9)	3.6 (2.2-5.9)	3.4 (2.3-5.4)	2.9 (2.0-4.3)	<.001
Albumin, median (IQR)	3.9 (3.7-4.2)	3.8 (3.5-4.1)	3.8 (3.5-4.1)	4.0 (3.8-4.2)	<.001
Sodium, median (IQR)	138.0 (136.0-139.0)	138.0 (135.0-140.0)	137.0 (135.0-139.0)	138.0 (136.0-140.0)	<.001
Diagnosis, No. (%)					<.001
>1 primary	363 (12)	46 (15)	116 (11)	201 (13)	
Breast	475 (16)	25 (8.3)	132 (13)	318 (20)	
Digestive system	476 (16)	51 (17)	201 (19)	224 (14)	
Endocrine system	27 (0.9)	2 (0.7)	13 (1.3)	12 (0.8)	
Female reproductive system	213 (7.3)	23 (7.6)	78 (7.6)	112 (7.1)	
Head and neck	75 (2.6)	5 (1.7)	26 (2.5)	44 (2.8)	
Hematologic system, No. (%)	184 (6.3)	29 (9.6)	69 (6.7)	86 (5.4)	
Integumentary system	102 (3.5)	8 (2.6)	34 (3.3)	60 (3.8)	
Lymphatic system	239 (8.2)	32 (11)	89 (8.6)	118 (7.4)	
Male reproductive system	183 (6.3)	8 (2.6)	47 (4.5)	128 (8.1)	
Nervous system	17 (0.6)	0 (11)	7 (0.7)	10 (0.6)	
Other	91 (3.1)	8 (2.6)	43 (4.2)	40 (2.5)	
Respiratory system	344 (12)	50 (17)	130 (13)	164 (10)	
Urinary system	133 (4.6)	15 (5.0)	48 (4.6)	70 (4.4)	
Treatment modality, No. (%)					
Platinum	1,188 (38)	130 (43)	442 (43)	546 (34)	<.001
Cytotoxic chemotherapy	1,741 (60)	198 (66)	665 (64)	878 (55)	<.001
Radiation therapy	249 (8.5)	36 (12)	90 (8.7)	123 (7.8)	.057
Oral chemotherapy	284 (9.7)	30 (9.9)	83 (8.0)	171 (11)	.068
Supportive care	73 (2.5)	6 (2.0)	32 (3.1)	35 (2.2)	.3
ADC	64 (2.2)	7 (2.3)	27 (2.6)	30 (1.9)	.5
Research	53 (1.8)	3 (1.0)	11 (1.1)	39 (2.5)	.018

NOTE. Predictors reflect the most recent value before chemotherapy initiation, obtained no more than 30 days before the regimen initiation. *P* values were calculated using Kruskal-Wallis tests for numeric variables and chi-square tests for categorical variables.

Abbreviations: ACU, acute care use; ADC, antibody-drug conjugates; A-ACU, all-cause acute care use; ECOG, Eastern Cooperative Oncology Group; P-ACU, preventable acute care use; RUCA, Rural-Urban Commuting Area.

#### Treatment Categorization

Cancer treatment regimens were categorized into therapeutic classes to examine their relationship with ACU. Categories included any cytotoxic chemotherapy, platinum-based chemotherapy, oral chemotherapy, immune checkpoint inhibitor, radiation therapy, hormonal therapy, bispecific T-cell engager, monoclonal antibody, antibody-drug conjugate, radiopharmaceutical, research study drug, supportive care, other, and multiple agent. Because regimens could contain agents from more than one class, patients could be assigned to multiple categories. For example, a regimen including both an oral chemotherapy agent and a hormonal therapy agent would be assigned three categories: oral chemotherapy, hormonal therapy, and multiple agent. Additional details are available in the Data Supplement (Table S2).

### Outcomes

ACU events within the health system were identified using the EMR and combined with events from linked insurance claims (approximately 9% of events) to capture care received outside the hospital system. We assessed six outcome measures: A-ACU and P-ACU at 30, 90, and 180 days after first cancer treatment infusion of a regimen. A-ACU was defined as any emergency room visit or hospitalization, regardless of reason for care. P-ACU, a subset of A-ACU, included visits with at least one discharge diagnosis identified by CMS as preventable. A summary of all models generated is presented in the Data Supplement (Fig S1).

### Statistical Methods

Statistical analyses were performed using R version 4.3.1 using *tidyverse* for data manipulation and visualization and *tidymodels* for model building.^26,27^ A two-sided *P* value <.05 was considered statistically significant.

### LASSO Logistic Regression Models

The data set was split into training (80%) and testing (20%) sets, stratified by outcome. Data preprocessing involved normalization of numeric predictors and one-hot encoding of categorical variables. Models were initially fit using the glmnet engine with a fixed penalty value. To optimize the penalty parameter, a grid search was conducted across 50 penalty values, with model performance assessed using stratified bootstrapping. The optimal penalty parameter was selected using the value that maximized the area under the receiver operating characteristic curve (ROC AUC). Model coefficients were scaled from –1 to +1. Model performances were evaluated on the test set, and performance metrics including ROC AUC, accuracy, precision, recall, and F1 score were reported. For each outcome, a separate optimal probability cutoff to classify whether a patient would experience ACU was determined by maximizing Youden's J index (*J* = sensitivity + specificity – 1) on the ROC curve. Patients were then stratified into high- and low-risk groups on the basis of this cutoff, and Kaplan-Meier curves were generated to estimate the cumulative incidence of ACU through 180 days after cancer treatment for each outcome. Differences between risk groups at 30, 90, and 180 days were assessed using log-rank tests.

### Random Forest Models

Data preprocessing and evaluation steps were identical to LASSO models. Models were fit using the ranger engine with 1,000 trees. A grid search was conducted across 25 combinations of the number of predictors sampled at each split (mtry) and the minimum node size (min_n). The optimal combination was selected on the basis of maximization of ROC AUC across 10-fold cross-validation on the training set. Predictor importance was estimated using permutation-based variable importance. As in the LASSO models, Youden's J index was used to determine outcome-specific cutoffs for stratifying patients into high- and low-risk groups, with Kaplan-Meier curves and log-rank tests comparing acute care utilization at 30, 90, and 180 days.

## RESULTS

### Cohort Characteristics

2,922 patients were included in the model that assessed ACU risk after first cancer treatment regimen initiation in the observation window. The median age was 68 years (IQR, 59-75), 52% were female, and 94% were Caucasian. Medicare was the most common primary insurance payer (51%), followed by commercial (40%), Medicaid (4.2%), self-pay (3.2%), and other (1.5%). RUCA classification identified 29% of patients as metropolitan, 31% micropolitan, 11% small town, and 29% rural (Table 1).

Patients were classified into three groups according to acute care utilization within 180 days of cancer treatment regimen initiation in Table 1: those having P-ACU, A-ACU without P-ACU, or no ACU. Across groups, significant differences were observed for age (*P* < .0001), sex (*P* = .031), race (*P* = .002), primary insurance payer (*P* < .0001), tumor type (*P* < .0001), weighted Charlson comorbidity index (*P* < .0001), number of previous inpatient admissions in the 6 months before cancer treatment regimen start (*P* < .0001), functional status (*P* < .0001), and smoking status (*P* = .012). Differences were also seen in laboratory values, including albumin (*P* < .0001), sodium (*P* < .0001), and NLR (*P* < .0001). In addition, cancer treatment modality also differed, including use of platinum therapy (*P* < .0001), cytotoxic therapy (*P* < .0001), and research study drugs (*P* < .0001).

Patients with P-ACU within 180 days of regimen initiation were older than those with A-ACU only or no ACU (median age 72 *v* 67 *v* 67 years, respectively), more frequently male (53% *v* 50% *v* 46%), and had greater comorbidity burden (≥8 weighted Charlson comorbidity index: 16% *v* 15% *v* 8.6%). They also had more hospitalizations in the 6 months before chemotherapy cancer treatment (≥2 admissions: 14% *v* 13% *v* 4.9%), poorer functional status (ECOG ≥2: 9.3% *v* 10% *v* 4.8%), and higher NLRs (median 3.6 *v* 3.4 *v* 2.9). Both the P-ACU and A-ACU groups had a higher proportion of patients with poor functional status (ECOG ≥2: 9.3% and 10% *v* 4.8%) and lower albumin levels compared with those with no ACU (3.8 and 3.8 vs 4.0 g/dL). Patients with P-ACU had increased rates of multiple primary tumors (15% *v* 11% *v* 13%) and respiratory cancers (17% *v* 13% *v* 10%), and lower rates of breast cancer (8.3% *v* 13% *v* 20%). Both the P-ACU and A-ACU groups had a higher proportion of patients with platinum-based (43% *v* 43% *v* 34%) and cytotoxic chemotherapy (66% *v* 64% *v* 55%), and lower proportion of patients with research study drug use (1.0% *v* 1.1% *v* 2.5%) than those without 180-day ACU (Table 1).

ACU rates varied by outcome type and time frame (Data Supplement, Table S3). A-ACU occurred in 45.3% of patients within 180 days, 32.3% within 90 days, and 16.1% within 30 days. P-ACU rates were lower, with 10.3% of patients affected within 180 days, 3.2% within 90 days, and 0.6% within 30 days.

**FIG 2.**
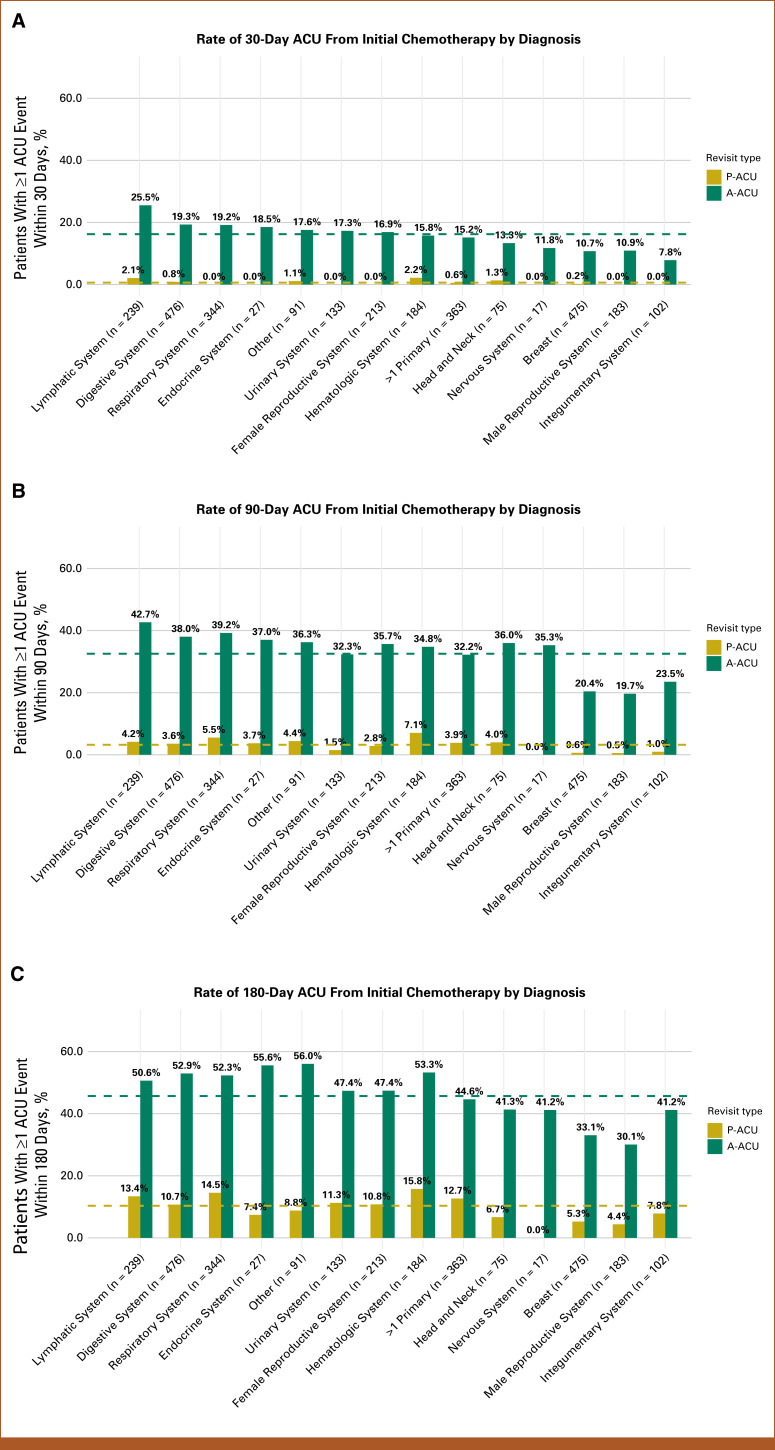
Proportion of patients with A-ACU (green) or P-ACU (yellow) ACU within (A) 30 days, (B) 90 days, and (C) 180 days of regimen initiation, by cancer type. Averages are indicated by dashed lines. ACU, acute care use; A-ACU, all-cause acute care use; P-ACU, preventable acute care use

As shown in Figure 2A, patients with lymphatic (25.5%) tumors had the highest 30-day A-ACU rates, while P-ACU rates were highest for those with hematologic (2.2%) and lymphatic (2.1%) tumors. At 90 days (Fig 2B), A-ACU rates were highest for patients with lymphatic (42.7%) and respiratory (39.2%) tumors, whereas P-ACU rates were highest for those with hematologic (7.1%) and respiratory (5.5%) tumors. By 180 days (Fig 2C), patients with other (56.0%) and endocrine (55.6%) tumors had the highest A-ACU rates, whereas patients with hematologic (15.8%) and respiratory (14.5%) tumors had the highest P-ACU rates.

### Modeling

#### Key Predictors

**FIG 3.**
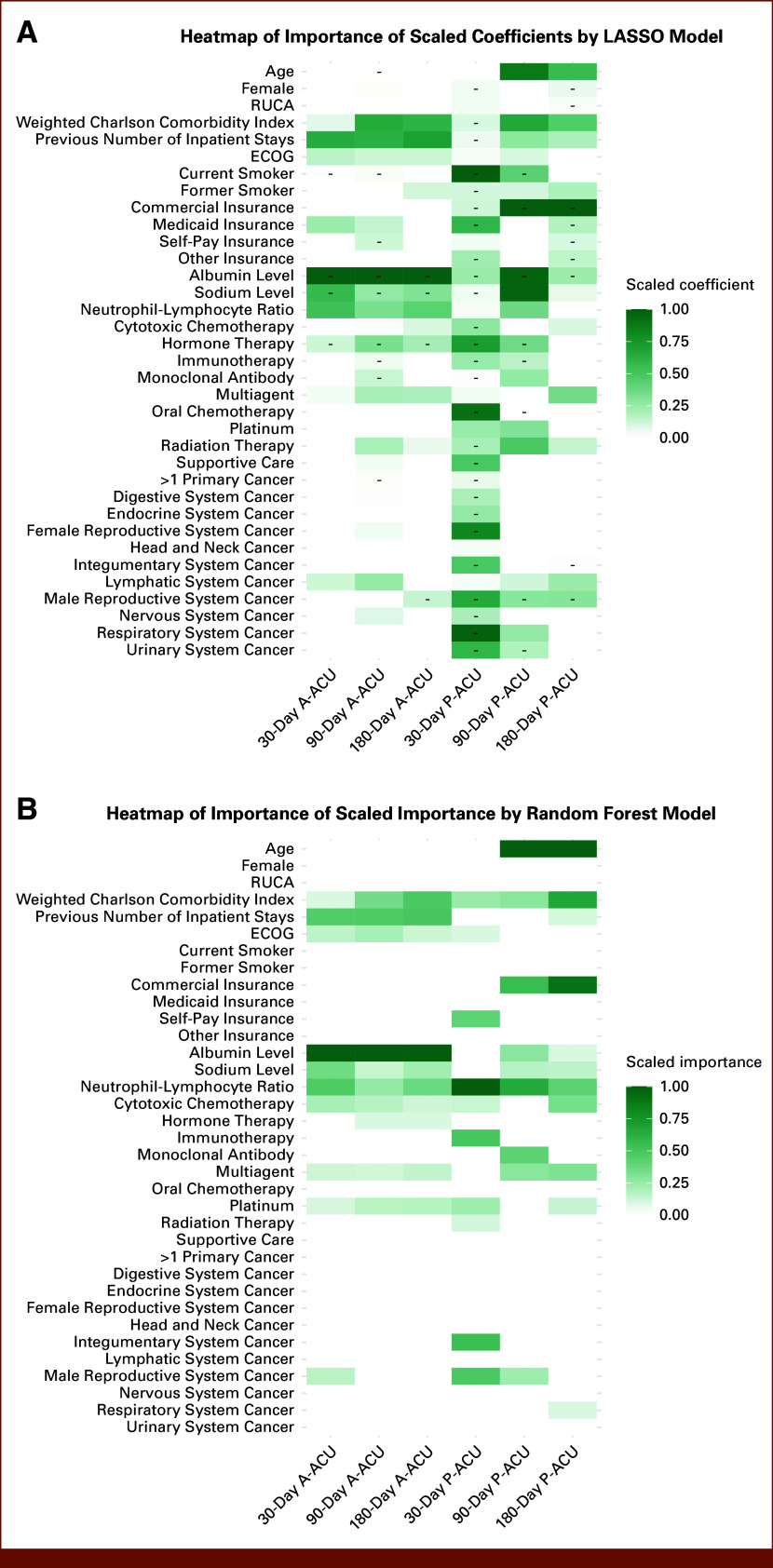
Heatmaps of scaled LASSO coefficients (A) and Random Forest importance (B) for predictors of A-ACU and P-ACU within 30, 90, and 180 days after chemotherapy regimen initiation. A-ACU, all-cause acute care use; ECOG, Eastern Cooperative Oncology Group; P-ACU, preventable acute care use; RUCA, Rural-Urban Commuting Area.

Across both LASSO and Random Forest models, weighted Charlson comorbidity index, number of previous inpatient stays, and NLR ranked among the most important predictors (Figs 3A and 3B; Data Supplement, Table S4). Insurance type and age were more influential in P-ACU models, while albumin was more strongly associated with A-ACU. Cancer type contributed minimally except for respiratory, lymphatic, and male reproductive system tumors in P-ACU models. Predictors were generally consistent for the 90- and 180-day models but differed for the 30-day P-ACU model, likely reflecting the low event rate (0.6% of patients).

#### Model Performance

Across outcomes, the models demonstrated strong predictive performance in assessing ACU risk from cancer treatment initiation (Table 2). Random Forest models produced ROC AUC values comparable with or slightly higher than those of LASSO models, while F1 scores were typically slightly higher for LASSO. Prediction thresholds derived from Youden's J index were highest for A-ACU models and consistently lower for P-ACU models, with both decreasing as the prediction window shortened. Because of substantial class imbalance in the 30-day P-ACU models (event rate: 0.6%), prediction thresholds were set at 0.01 for both LASSO and Random Forest models. As a result, the findings from these models should be interpreted with caution.

**TABLE 2.** LASSO and Random Forest Model Performance Metrics for Predicting A-ACU or Preventable P-ACU Within 30, 90, and 180 days of Chemotherapy Regimen Initiation

Model	LASSO						Random Forest					
	Threshold	AUC	Accuracy	Precision	Recall	F1	Threshold	AUC	Accuracy	Precision	Recall	F1
30-day A-ACU	0.16	0.75	0.71	0.92	0.72	0.81	0.18	0.77	0.77	0.92	0.79	0.85
90-day A-ACU	0.34	0.74	0.71	0.80	0.76	0.78	0.33	0.74	0.68	0.84	0.66	0.74
180-day A-ACU	0.48	0.66	0.64	0.65	0.75	0.69	0.48	0.68	0.65	0.66	0.71	0.69
30-day P-ACU	0.01	0.66	0.71	1.00	0.71	0.83	0.01	0.73	0.59	1.00	0.59	0.74
90-day P-ACU	0.05	0.72	0.81	0.99	0.82	0.90	0.04	0.73	0.76	0.99	0.77	0.86
180-day P-ACU	0.13	0.75	0.72	0.96	0.72	0.82	0.11	0.77	0.63	0.98	0.60	0.74

Abbreviations: A-ACU, all-cause acute care use; P-ACU, preventable acute care use.

**FIG 4.**
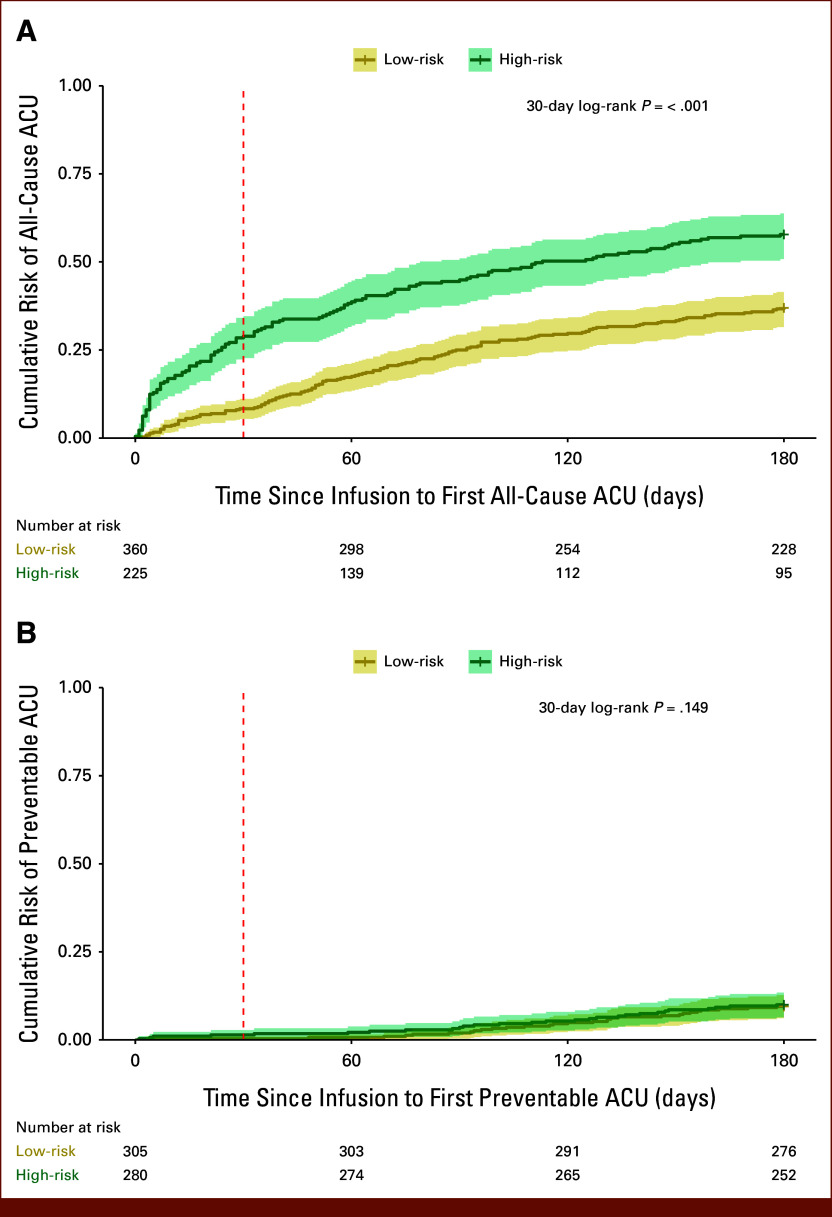
Kaplan-Meier plots showing the predicted probability of (A) A-ACU and (B) P-ACU over time after chemotherapy regimen initiation from the respective 30-day models. The *y* axis represents the predicted probability of ACU and the *x* axis shows time in days from regimen initiation. ACU, acute care use; A-ACU, all-cause acute care use; P-ACU, preventable acute care use.

The 30-day LASSO model Kaplan-Meier curves for A-ACU and P-ACU are shown in Figures 4A and 4B, respectively. The corresponding 30-day Random Forest curves, as well as all 90- and 180-day LASSO and Random Forest curves, are provided in the Data Supplement (Figs S2-S6A, B). There was significant separation between 30-day A-ACU risk groups (*P* < .001; Fig 4A; Data Supplement, Fig S2A). Although differences between P-ACU risk groups in the random forest models were also significant (LASSO *P* = .149, Random Forest *P* = .017; Fig 4B; Data Supplement, Fig S2B), risk group trajectories crossed after the 30-day mark. The models for 90- and 180-day A-ACU and P-ACU demonstrated consistent separation between groups, with no crossing of risk trajectories (all *P* < .001; Data Supplement, Figs S3–S6A, B).

## DISCUSSION

In this study, 45.3% of patients experienced an A-ACU event within 180 days of cancer treatment initiation and 10.3% experienced a P-ACU event—rates that are slightly lower but generally consistent with previous literature.^4^ To address this, we predicted A-ACU and P-ACU risk after the initial infusion of a new regimen and identified modifiable risk factors and high-risk patients using routinely collected EMR data with strong discrimination across 30-, 90-, and 180-day time frames.

Key predictors of ACU included previous hospitalizations, comorbidity burden, and inflammatory/nutritional biomarkers (NLR, sodium, and albumin), suggesting patients with recent hospitalizations, systemic inflammation, and compromised nutritional status are at higher risk. The associations are consistent with previous literature linking previous hospitalizations, comorbidities, and increased risk of ACU.^17,19,20^ Similarly, lower albumin levels have been linked to higher risk of ACU.^17,19,21,22^ Cancer type contributed minimally to model performance, suggesting patient-level factors and underlying health status are more influential drivers of ACU than specific tumor characteristics. Older adults and Medicare enrollees had higher P-ACU risk, pointing to the influence of access to care and socioeconomic factors, and further underscoring the need for interventions that address health care disparities. Further investigation is warranted, as previous studies have shown mixed findings regarding age.^4,18^ Unlike previous studies in nononcology populations,^28^ we did not find a significant association between rurality and ACU, although rural patients may still be at increased risk of ACU because of limited access to specialist care and longer travel times.

Several limitations should be considered. The low frequency of 30-day P-ACU events (0.6%) resulted in class imbalance, requiring cautious interpretation. Additionally, reliance on linked claims to supplement system-specific EMR ACU data may have led to an underestimation of P-ACU as claims captured only the primary diagnosis code, and in some cases a secondary diagnosis could have reclassified the ACU as preventable. Finally, the retrospective design and rural cohort may limit the generalizability of our findings.

Although our findings did not establish a direct link between rurality (RUCA) and ACU, further research into the prevalence of risk factors for ACU in rural populations is warranted.

In conclusion, this work advances ACU risk prediction during cancer treatment by identifying and validating key predictors and offering new insights into how predictors differentially affect A-ACU versus P-ACU. Our findings confirm the importance of considering patient-level factors, including comorbidities, socioeconomic status, and inflammatory/nutritional markers when accessing risk and individualizing care. By integrating these models into clinical practice and addressing the unique challenges faced by rural patients, we can potentially reduce ACU, improve outcomes, and deliver better care.
